# Selective Biological Effects of Selenium-Enriched Polysaccharide (Se-Le-30) Isolated from *Lentinula edodes* Mycelium on Human Immune Cells

**DOI:** 10.3390/biom11121777

**Published:** 2021-11-26

**Authors:** Beata Kaleta, Aleksander Roszczyk, Michał Zych, Monika Kniotek, Radosław Zagożdżon, Marzenna Klimaszewska, Eliza Malinowska, Michał Pac, Jadwiga Turło

**Affiliations:** 1 Department of Clinical Immunology, Medical University of Warsaw, Nowogrodzka 59, 02-006 Warsaw, Poland; aleksander.roszczyk@wum.edu.pl (A.R.); michal.zych@wum.edu.pl (M.Z.); monika.kniotek@wum.edu.pl (M.K.); radoslaw.zagozdzon@wum.edu.pl (R.Z.); 2 Department of Drug Technology and Pharmaceutical Biotechnology, Medical University of Warsaw, Banacha 1, 02-097 Warsaw, Poland; marzenna.klimaszewska@wum.edu.pl (M.K.); eliza.malinowska@wum.edu.pl (E.M.); jadwiga.turlo@wum.edu.pl (J.T.); 3 Department of Immunology, Transplantology, and Internal Diseases, Medical University of Warsaw, Nowogrodzka 59, 02-006 Warsaw, Poland; michal.pac@student.wum.edu.pl

**Keywords:** *Lentinula edodes*, selenium, polysaccharide, immunosuppressant

## Abstract

A common edible mushroom *Lentinula edodes,* is an important source of numerous biologically active substances, including polysaccharides, with immunomodulatory and antitumor properties. In the present work, the biological activity of the crude, homogenous (Se)-enriched fraction (named Se-Le-30), which has been isolated from *L. edodes* mycelium by a modified Chihara method towards human peripheral blood mononuclear cells (PBMCs) and peripheral granulocytes, was investigated. The Se-Le-30 fraction, an analog of lentinan, significantly inhibited the proliferation of human PBMCs stimulated with anti-CD3 antibodies or allostimulated, and down-regulated the production of tumor necrosis factor (TNF)-α by CD3+ T cells. Moreover, it was found that Se-Le-30 significantly reduced the cytotoxic activity of human natural killer (NK) cells. The results suggested the selective immunosuppressive activity of this fraction, which is non-typical for mushroom derived polysaccharides.

## 1. Introduction

Research over the past twenty years has shown that mushrooms are a valuable source of numerous bioactive substances [1]. The best-known components with documented immunomodulatory and antitumor properties are mushroom-derived polysaccharides [2,3,4]. It has been demonstrated that these compounds can affect T and B lymphocytes, natural killer (NK) cells, macrophages, and neutrophils [3,5]. The immunomodulatory properties of fungal polysaccharides depend on their structure, in particular their monosaccharide composition, molecular weight, branching degrees, presence of functional groups, water-solubility, as well as their triple-helical conformation [6]. Lentinan is one of the best known mushroom-derived compounds. It is a highly purified (1-3;1-6)-β-d-glucan, extracted from *Lentinula edodes* (shiitake mushroom) fruiting bodies. Lentinan is used in China, Japan, and Russia as an anticancer drug and is usually administered with basic therapy [7,8]. It is postulated that the anticancer activity of this glucan is associated with its immunostimulatory properties and not with the direct impact on tumor cells [9,10].

Selenium (Se) is a vital micronutrient, important for various aspects of human health, including immune responses. Se plays a significant role in the activation, proliferation, and differentiation of immune cells [11,12,13,14]. Therefore, in our previous studies, we have synthesized, isolated, and characterized an Se-containing analog of lentinan and examined whether Se incorporation affects its biological activity. Structural studies have shown that the isolated fraction was a protein-containing mixture of high molecular weight polysaccharides, α and β-glucans containing glycosidically-bonded Se [15]. Regardless of the Se content, the structure of the isolated fraction was dissimilar to lentinan [15]. Next, we have analyzed the impact of this lentinan analog on cells viability, its antioxidant activity, effects on human peripheral blood mononuclear cells (PBMCs) proliferation, and the production of reactive oxygen species by granulocytes [16]. Our analyses implicated selective immunosuppressive activity, untypical for fungal polysaccharides, that was further potentiated by Se incorporation. However, it was not clear which component of this fraction was responsible for the observed immunosuppressive effects. Thus, the goal of the current work was to obtain a purer and more homogenous fraction from the Se-enriched mycelium of *L. edodes* using the modified Chihara method [17], to investigate the biological activity of this Se-enriched extract (named Se-Le-30) in human immune cells in vitro.

## 2. Materials and Methods

### 2.1. Biosynthesis and Extraction of Se-Enriched Polysaccharide Fraction (Se-Le-30)

The *Lentinula edodes* (Berk.) Pegler strain used in our study was ATCC 48085 (ATCC, Manassas, VA, USA). The culture medium was fortified with selenium (Se) at a concentration of 30 μg/mL by the addition of sodium selenite (Sigma, Saint Louis, MO, USA). Se-enriched mycelia of *L. edodes* were cultivated under the submerged conditions described in our previous papers [15,18,19,20]. Mycelia were harvested by filtration, washed three times with distilled water, and freeze-dried.

The Se-enriched polysaccharide fraction (Se-Le-30), an analog of the Japanese anticancer drug lentinan, was isolated from the Se-enriched *L. edodes* mycelium via the modified Chihara method [17]. We have completed the original method by the preliminary extraction of lipids, small carbohydrate molecules, and other non-polysaccharide compounds, with methanol (4 h of extraction in boiling methanol, 1:4 *w/v*).

### 2.2. Se-Le-30 Fraction Structure Examination

The Se content, monosaccharide composition, protein content, and type of the glycosidic bonds were determined by methods described in our previous paper [15]. Briefly, the Se content was determined by the modified fluorometric method (RP HPLC with fluorescence detection), the monosaccharide composition of the polysaccharide was determined by the use of reversed-phase high-performance liquid chromatography (RP HPLC), the protein content was determined by the use of the Bradford method, verified was determined via the measurement of the nitrogen content in the analyzed fractions, and the type of glycosidic bonds were determined by spectral analysis [20,21].

### 2.3. The Analysis of the Biological Activity of Se-Le-30

#### 2.3.1. The Effects of Se-Le-30 on Mitogen-Stimulated Human Peripheral Blood Mononuclear Cells (PBMCs) Proliferation

PBMCs from 15 healthy donors were separated by density gradient centrifugation on Histopaque-1077 (Sigma, Saint Louis, MO, USA). Samples were commercially obtained from the Regional Blood Centre in Warsaw. PBMCs (1 × 10^6^ cells/well) were cultured in 96-well flat-bottom microplates in a RPMI 1640 medium (Gibco, Thermo Fisher Scientific, Waltham, MA, USA) containing 2 mM L-glutamine (Sigma, Saint Louis, MO, USA), antibiotic-antimycotic solution (1.5% penicillin-streptomycin-amphotericin, Invitrogen, Thermo Fisher Scientific, Waltham, MA, USA), and 10% fetal bovine serum (FBS, Gibco, Thermo Fisher Scientific, Waltham, MA, USA). PBMCs were stimulated with mitogens: anti-CD3 mAb (OKT3, 1 μg/mL, BD Pharmingen, San Diego, CA, USA), phytohemagglutinin (PHA, 20 μg/mL, Sigma, Saint Louis, MO, USA), or suspension of Staphylococcus aureus Cowan strain (SAC, 0.004% *w/v*, Calbiochem, San Diego, CA, USA), and incubated with the Se-Le-30 fraction in concentrations of 1, 10, and 100 µg/mL to repeat the pattern of our previous experiments [16]. As an additional control, an analogous test without any mitogens (autostimulation) was carried out. PBMCs were cultured for 72 h at 37 °C in a humidified atmosphere with 5% CO_2_. Cells were pulsed with a 1 μCi/well of [3H]-thymidine (113 Ci/nmol, NEN) for 18 h before the end of culture. When culture ended, cells were harvested, and the radioactivity was measured with a liquid scintillation counter (Wallac, PerkinElmer, Waltham, MA, USA), giving the level of radioactivity as ‘corrected counts per minute’ (ccpm). All experiments were performed in triplicates.

#### 2.3.2. The Effects of Se-Le-30 on Alloantigen-Stimulated Human Peripheral Blood Mononuclear Cells (PBMCs) Proliferation in Mixed Lymphocyte Reaction (MLR)

PBMCs were isolated from the heparinized blood of 10 donors, as described above, and resuspended in a RPMI 1640 medium (Gibco, Thermo Fisher Scientific, Waltham, MA, USA) containing 2 mM L-glutamine (Sigma, Saint Louis, MO, USA), antibiotic-antimycotic solution (1.5% penicillin-streptomycin-amphotericin, Invitrogen, Thermo Fisher Scientific, Waltham, MA, USA), and 10% fetal bovine serum (FBS, Gibco, Thermo Fisher Scientific, Waltham, MA, USA). PBMCs were divided into two groups: (1) responder cells and (2) stimulatory cells, which were inactivated by gamma-irradiation for 90 min. The MLR was performed via the co-culture of 1 × 10^5^ of responder cells and 1 × 10^5^ of stimulatory cells. PBMCs were treated with Se-Le-30 at a concentration of 100 µg/mL. Control cultures contained an equivalent volume of the medium. PBMCs cultures were incubated at 37 °C in 5% CO_2_ in a humidified atmosphere for 72 h. DNA synthesis was measured in the last 18 h by adding a 1 μCi/well of [3H]-thymidine (113 Ci/nmol, NEN, PerkinElmer, Waltham, MA, USA). Cells were harvested, and [3H]-thymidine uptake (expressed as radioactivity, ccpm) was quantified in a scintillation counter (Wallac PerkinElmer, Waltham, MA, USA). All experiments were performed in triplicates.

#### 2.3.3. The Effects of Se-Le-30 on Cytotoxicity of Natural Killer (NK) Cells

PBMCs were isolated from 11 blood donors by density-gradient centrifugation, as described above, and adjusted to 1 × 10^7^ cells/2.6 mL in culture medium (RPMI 1640 with 10% FBS). A human chronic leukemia cell line, K562 (ATCC #CCL-243), was used as the target cell line. K562 cells were stained with 1.2 µL 3,3-dioctadecyloxacarbocyanine perchlorate (Sigma) for 20 min at 37 °C in a sterile environment with 5% CO_2_ and a humidified atmosphere. After incubation, the cells were suspended in a RPMI 1640 medium (with 10% FBS) to a concentration of 1 × 10^6^ cells/mL and used for the cytotoxicity assay. Effector cells were incubated for 4 h at 37 °C with target cells in the following combinations: PBMCs in RPMI, K562 in RPMI, PBMCs with K562 in ratios of 50:1 and 12:1, and PBMCs with K562 in ratios of 50:1 and 12:1 supplemented with Se-Le-30 in the concentrations of 1, 10 and 100 μg/mL. After incubation, the cells were stained with propidium iodide solution (0.1 μg/mL, Sigma) to detect dead cells. Live target cells (T- K562 cells) were identified as cells with low fluorescence in a channel dedicated to PE (negative for propidium iodide) and with positive, above the third decade on a logarithmic scale, in a channel dedicated for FITC (positive for 3.3-dioctadecyloxacarbocyanine perchlorate). Dead target cells (Td- K562) were distinguished as with positive fluorescence in both FITC and PE channels. The percentage of dead target cells was calculated as follows: %Td = (Td/T) × 100. Specific lysis was calculated by %Td cultured with effector cells—%Td cultured without effector cells. Cytotoxicity against K562 was analyzed using the FACS Canto II Flow Cytometer (BD Biosciences, San Jose, CA, USA) and BD FACS Diva 6.1.3. software.

#### 2.3.4. The Effects of Se-Le-30 on TNF-α, IFN-γ, TGF-β, and IL-10 Production by CD3+ T Cells

PBMCs from healthy donors were isolated, as described above, and suspended at a density of 1 × 10^6^ cells/mL in a RPMI 1640 medium supplemented with 10% heat-inactivated fetal calf serum (FCS, Sigma), 2 mM glutamine (Sigma), and antibiotic-antimycotic solution (100 I.U. penicillin, 100 μg/mL streptomycin and 0.25 μg/mL amphotericin, Corning). Cells were cultured in 24-well plates (Nunc, Thermo Fisher, Waltham, MA, USA) and incubated for 48 h (37 °C, 5% CO_2_) in the presence of Se-Le-30 in a concentration of 100 µg/mL, and separately with the same volume of 0.9% NaCl (Fresenius Kabi, Bad Homburg, Germany) as a control. After incubation, cells were stimulated with 50 ng/mL PMA (Sigma, Saint Louis, MO, USA) and 1 μg/mL ionomycin (Sigma, Saint Louis, MO, USA), in the presence of 4 μL/mL Golgi-Stop (BD Biosciences, San Jose, CA, USA) for 4 h. Then, cells were washed twice and resuspended in 100 μL stain buffer (BD Biosciences, San Jose, CA, USA), stained with monoclonal antibody anti-CD3, PerCP (SK7-clone, BD Biosciences, San Jose, CA, USA) for 15 min, at room temperature in the dark. After surface antigen staining, cells were washed in 1000 µL, then resuspended in 300 μL BD Cytofix/Cytoperm solution (BD Biosciences, San Jose, CA, USA) for 20 min at 4 °C for permeabilization. In the next step, cells were washed twice in 1 mL and resuspended in 100 μL BD Perm/Wash™ buffer, then monoclonal antibodies against intracellular cytokines were added: anti-IFN-γ PE-Cy7 (B27 clone, BD Biosciences, San Jose, CA, USA), anti-TNF-α FITC (MAb11 clone, BD Biosciences, San Jose, CA, USA), anti-IL-10 PE (JES3-9D7 clone, Becton Dickinson), and anti-TGF-β PE (TW4-9E7 clone, BD Biosciences, San Jose, CA, USA), incubated in darkness for 30 min at 4 °C, washed once in 1 mL Perm/Wash buffer and resuspended in 300 μL of stain buffer. Cell readouts data was acquired using a Becton Dickinson FACS Canto II cytometer (BD FACS Canto II, BD Biosciences, San Jose, CA, USA) and analyzed with BD FACS Diva 6.1.3 software. Analyses were conducted on live cells.

#### 2.3.5. The Effects of Se-Le-30 on Superoxide Production by Granulocytes

Granulocytes were separated from whole blood samples of six healthy donors (commercially obtained from the Regional Blood Centre in Warsaw) by density gradient centrifugation on Histopaque-1077 and Histopaque-1119 (Sigma, Saint Louis, MO, USA). Granulocytes were harvested from the interface between Histopaque-1077 and Histopaque-1119 and washed two times with cold phosphate-buffered saline (PBS, Biomed, Lublin, Poland). Granulocytes (2.5 × 10^5^ cells/well) were cultured in 96-well round-bottom microplates in a sterile medium containing PBS, 6 mM glucose (Sigma, Saint Louis, MO, USA), and 1% bovine serum albumin (BSA, Biowest, Nuaillé, France). Granulocytes were either activated or not by phorbol 12-myristate 13-acetate (PMA, Sigma, Saint Louis, MO, USA) and incubated with cytochrome c (Sigma, Saint Louis, MO, USA) and Se-Le-30 in the concentration of 100 μg/mL for 30 min at 37 °C in a humidified atmosphere with 5% CO_2_. The concentration of 100 μg/mL was chosen because it was the highest active concentration of polysaccharides tested in our previous studies [16]. The number of superoxide anions (nmols O_2_^−^) generated by granulocytes was calculated using the Lambert–Beer law. Control cultures contained an equivalent amount of medium. To correct O_2–_ independent reduction of cytochrome c, an additional control with superoxide dismutase (SOD, 30 mg/mL, Sigma, Saint Louis, MO, USA) was used. Generation of O_2–_ by reduction of cytochrome c was detected at room temperature using a microplates reader at 550 nm (Chromate 4300 Microplate Reader, BioFront Technologies, FL, USA). All experiments were performed in triplicates.

### 2.4. Statistical Analysis

Data gathered from the experiment was tested for normality distribution with the Shapiro–Wilk and Lilliefors tests. The Wilcoxon paired test was used for data series that did not have a normal distribution. The Student *t*-test was performed for the series with normal distribution, confirmed with both of the normality tests mentioned. ANOVA was used for data sets with normal distribution, and the Friedman test for data series with at least one data set did not show normal distribution, and data failed to be transformed to normal distribution. The data were analyzed with Statistica 13.1. Figures were prepared with GraphPad Prism 7. A probability value of *p* < 0.05 with a 95% confidence interval was considered to indicate a statistically significant difference.

## 3. Results

### 3.1. Structural Analysis

A detailed structural analysis of the Se-Le-30 being tested in the current work (including the first-order structure of its polysaccharide components) has been described in a separate publication [18]. To avoid repeating the results presented there, we will give only brief information on the composition of this fraction, as stated below.

The yield of the extraction and purification of Se-Le-30 by the modified Chihara method was low and amounted to about 0.04% of the mycelial dry weight. The concentration of Se was 48 µg/g. The total content of proteins was 3.3%. Glucose and mannose constituted nearly 96.7% of total monosaccharides, in a proportion of approximately 93:4 by mass. Se-Le-30 turned out to be a mixture of four polysaccharide structures, none of which is similar are lentinan (the structure of polysaccharides presented in fraction Se-Le-30 versus lentinan are presented in Supplementary Figure S1). Importantly, these structures were difficult to separate from each other due to intermolecular interactions. As shown in our previous paper [18], the main component of the Se-Le-30 fraction was a linear 1-4-α-d-glucan with an Mw > 2000 kDa, with probable helical structure, and was much more flexible and hydrophilic than lentinan (A). The next component of the Se-Le-30 fraction was a mixture of unbranched 1-6-β-d-glucan, unbranched 1-3-β-d-glucan, and 1-3-β-branched 1-6-β-d-glucan—all with a probable helical conformation (B, C and D). As we stated in our previous work [18], the content of the polysaccharides B-C/D in the Se-Le-30 fraction was less than 3% which suggested its low content in the *L. edodes* cell wall (or low extractability with hot water).

The molar mass of the analyzed polysaccharide was: M_n_ = 1.69 × 10^6^ g/mol, M_w_ = 3.62 × 10^6^ g/mol, Ð = 2.15 (dispersity).

### 3.2. Biological Activity of Se-Le-30

#### 3.2.1. The Effect of Se-Le-30 on Proliferation of the Mitogen-Stimulated Human Peripheral Blood Mononuclear Cells (PBMCs)

**Figure 1:**
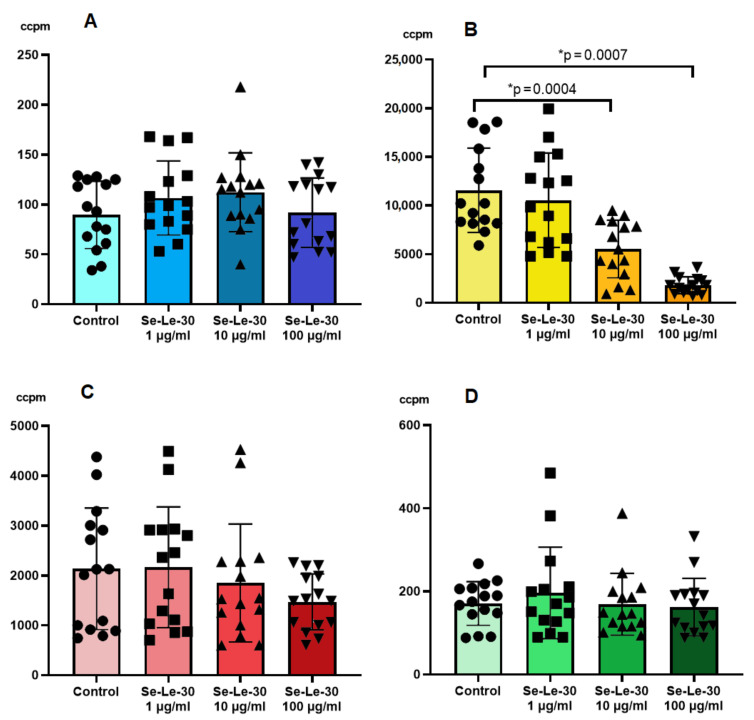
The effect of Se-Le-30 (in the concentrations of 1, 10 and 100 µg/mL) on the proliferation of human peripheral blood mononuclear cells (PBMCs) non-stimulated (**A**), stimulated with anti-CD3 monoclonal antibody (OKT3, **B**), phytohemagglutinin (PHA, **C**) or suspension of Staphylococcus aureus Cowan strain (SAC, **D**). The proliferation of lymphocytes was examined on the DNA synthesis level by the measurements of 3H-thymidine incorporation. The results are presented as a level of radioactivity measured as ‘corrected counts per minute’ (ccpm). Statistical differences were considered when *p* < 0.05. The mean value (*n* = 15) and standard deviation are given. * *p* < 0.05 vs. control.

The mitogen-induced proliferation of PBMCs is a widely used assay to assess non-specific cellular immunity. To study this response, human PBMCs were stimulated with two T-cells mitogens (anti-CD3 mAb (OKT3) and PHA) or a suspension of Staphylococcus aureus Cowan strain (SAC, B cells mitogen). The effect of Se-Le-30 on the mitogen-stimulated proliferation of human PBMCs is presented in Figure 1. The study demonstrated that Se-Le-30 in the concentrations of 10 and 100 µg/mL significantly inhibited OKT3-induced proliferation of PBMCs (*p* = 0.0004 and *p* = 0.0007, respectively). However, no significant inhibitory effect of this fraction on PHA- and SAC-stimulated PBMCs was detected.

The cell viability assessment via the trypan blue exclusion method indicated that the inhibitory effect on OKT3-stimulated PBMCs proliferation was not due to the toxicity of the analyzed polysaccharide.

#### 3.2.2. The Effect of Se-Le-30 on Alloantigen-Stimulated Human Peripheral Blood Mononuclear Cells (PBMCs) Proliferation in Mixed Lymphocyte Reaction (MLR)

Mixed lymphocyte reaction (MLR) has been used for the selection of donors for transplantation. Today the MLR-based assay is an efficient model for the study of T-cell activation and proliferation, and for the monitoring of the immune status of transplant organ recipients. In MLR, T cells from two unrelated donors are cocultured. The foreign histocompatibility antigens present on allogeneic cells serve as stimulators of proliferation.

**Figure 2:**
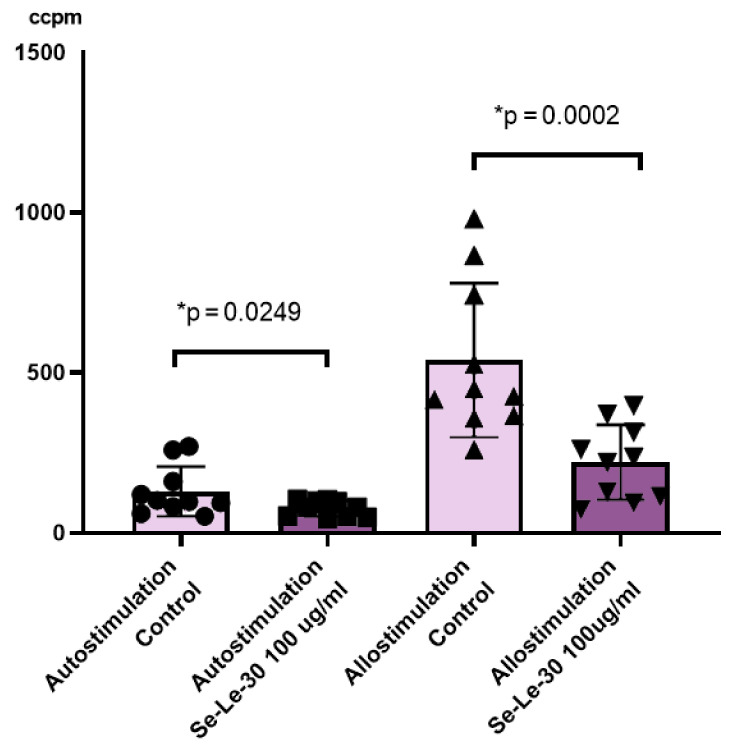
The effect of Se-Le-30 (100 μg/mL) on peripheral blood mononuclear cells (PBMCs) proliferation in a mixed lymphocyte reaction (MLR). The proliferation of lymphocytes cultured with irradiated PBMCs from the same donor (autostimulation) or from the second unrelated donor (allostimulation) was examined on the DNA synthesis level by the measurement of 3H-thymidine incorporation. The results are presented as a level of radioactivity measured as ‘corrected counts per minute’ (ccpm). Statistical differences were considered when *p* < 0.05. The mean value (*n* = 10) and standard deviation are given. * *p* < 0.05 vs. control.

The effect of Se-Le-30 on auto- and allo-stimulated PBMCs proliferation is presented in Figure 2. It was demonstrated that this fraction significantly inhibited a proliferative response in the MLR assay performed with human auto- or allo-stimulated PBMCs (*p* = 0.0249 and *p* = 0.0002, respectively).

#### 3.2.3. The Effect of Se-Le-30 on Cytotoxicity of Natural Killer (NK) Cells

**Figure 3:**
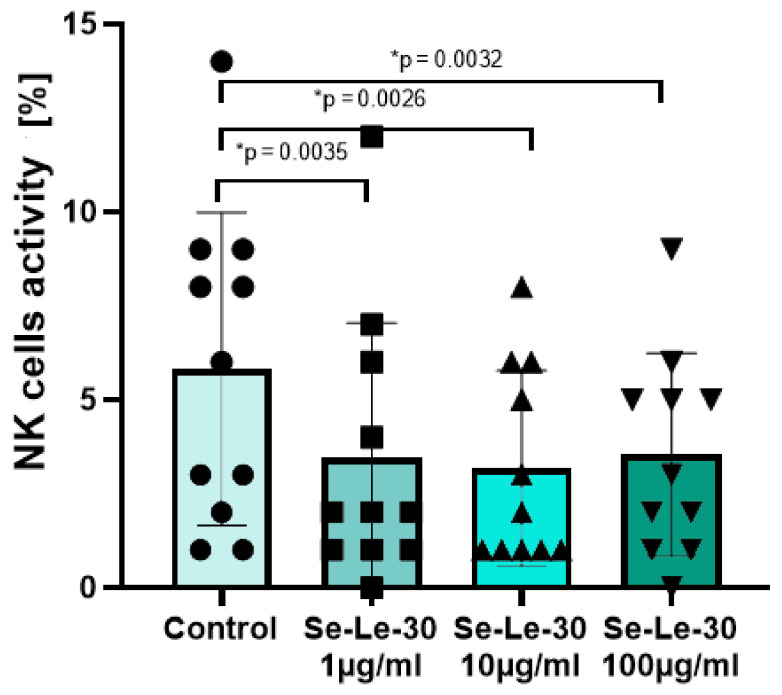
The effects of Se-Le-30 (in concentrations of 1, 10 and 100 µg/mL) on the cytotoxic activity of human natural killer (NK) cells. Statistical differences were considered when *p* < 0.05. The mean value (*n* = 11) and standard deviation are given. * *p* < 0.05 vs. control.

The effect of Se-Le-30 on the cytotoxic activity of human NK cells is presented in Figure 3. The study demonstrated that this Se-enriched polysaccharide significantly down-regulated the cytotoxicity of NK cells in the concentrations of 1, 10, and 100 µg/mL (*p* = 0.0039, *p* = 0.0039, and *p* = 0.0032, respectively).

#### 3.2.4. The Effect of Se-Le-30 on Tumor Necrosis Factor (TNF)-α, Interleukin (IL)-10, Interferon (INF)-γ and Transforming Growth Factor (TGF)-β Production by CD3+ T Cells

**Figure 4:**
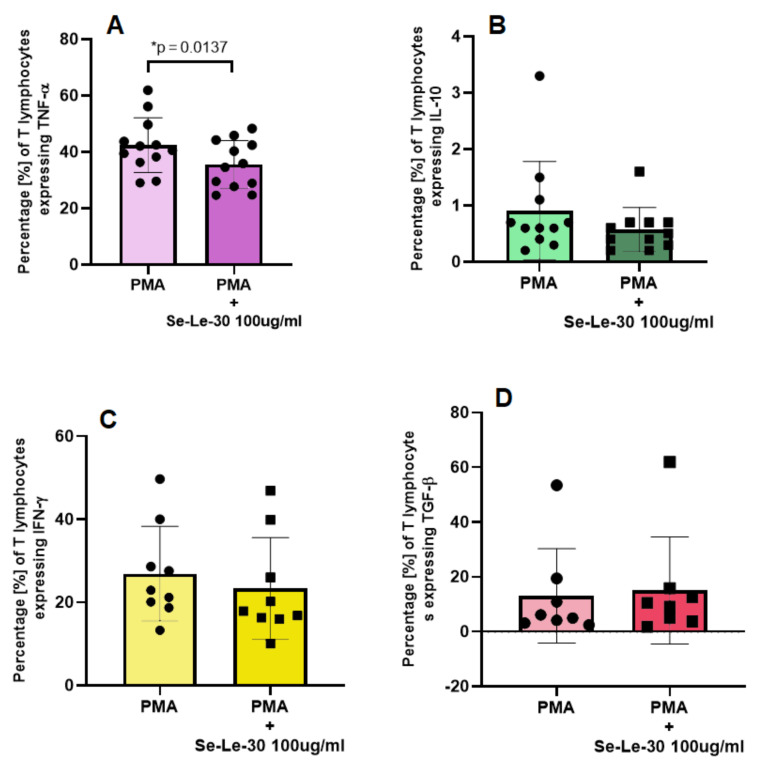
The effects of Se-Le-30 (100 µg/mL) on the percentage of CD3+ T lymphocytes producing tumor necrosis factor (TNF)-α (**A**), interleukin (IL)-10 (**B**), interferon (IFN)-γ (**C**), and transforming growth factor (TGF)-β (**D**). Statistical differences were considered when *p* < 0.05. The mean values (*n* = 12 for TNF-α, *n* = 11 for IL-10, *n* = 9 for IFN-γ, and *n* = 8 for TGF-β) and standard deviations are given. * *p* < 0.05 vs. control. For a detailed explanation of the gating strategy, please see Supplementary Figure S2.

The effect of 100 µg/mL of Se-Le-30 on TNF-α, IL-10, INF-γ, and TGF-β production by CD3+ T cells is presented in Figure 4. The study demonstrated that Se-Le-30 significantly down-regulated the number of TNF-α producing CD3+ T cells, however, did not affect the number of IL-10, IFN-γ and TGF-β producing CD3+ T cells.

#### 3.2.5. The Effect of Se-Le-30 on Superoxide Anion (O_2-_) Production by Granulocytes

**Figure 5:**
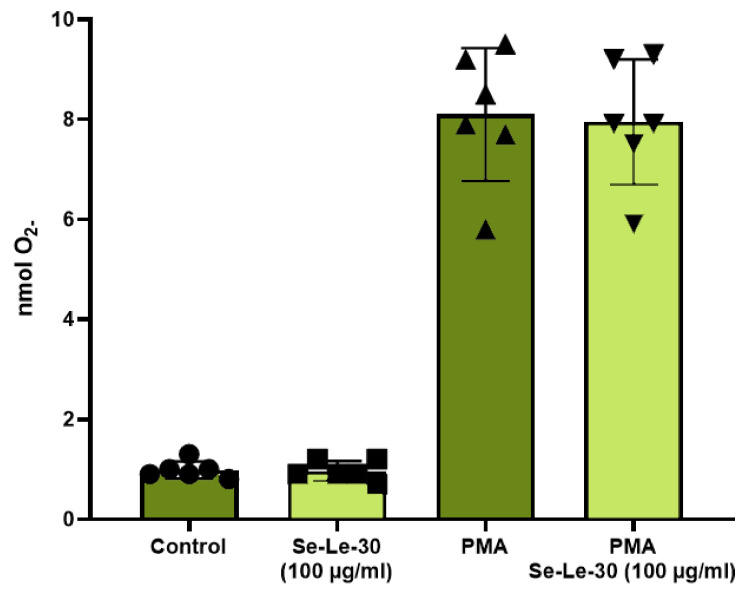
The effect of Se-Le-30 (in the concentration of 100 µg/mL) on the production of superoxide anions (O_2-_) by human peripheral blood granulocytes. The auto- and phorbol 12-myristate 13-acetate (PMA) stimulation of granulocytes is presented as a concentration of O_2−_ determined by cytochrome c reduction rate. *p* value was calculated for control cultures vs. cultures with Se-Le-30 and PMA stimulated cultures vs. cultures with PMA and Se-Le-30. The mean value (*n* = 6) and standard deviation are given.

Granulocytes generate reactive oxygen species, including superoxide anion (O_2-_), during phagocytosis and in response to soluble agonists. To study this response, the production of O_2-_ in the cytochrome c reduction assay was measured. The analysis demonstrated that Se-Le-30 has no significant effect on the O_2−_ production by human granulocytes (*p* = 0.715) (Figure 5).

## 4. Discussion

In the last decades, numerous mushroom-derived polysaccharides have been described and used as immunomodulatory and anti-cancer agents [2,9,10]. The exact mechanism of their action is not fully explained, but it was revealed that they act as biological response modifiers (BRMs) that stimulate cell-mediated immune response [9,10]. One of the best-characterized polysaccharides of fungal origin is lentinan, isolated from fruiting bodies of *L. edodes*. Due to their unique properties and confirmed safety, fungal polysaccharides, mainly β-glucans from *L. edodes*, are used in the prevention and adjuvant therapy in many diseases [7,8].

In our previous studies, we used the Yap and Ng [22] method for the isolation of the polysaccharide fractions from the Se-enriched mycelium of *L. edodes*. This method is known to be considerably milder than a multi-step fractionation procedure used for isolation of lentinan from fruiting bodies of *L. edodes* by Chihara [17]. In accordance with our expectations, the currently studied polysaccharide (named Se-Le-30), isolated by modified Chihara method from the Se-enriched mycelium of *L. edodes*, was characterized by a higher purity but much lower content of Se as compared to described in our earlier papers fraction Se-L [15,16].

As mentioned above, the immunomodulatory properties of mushroom-derived polysaccharides depend on their structure [6]. Lentinan, one of the best known natural enhancers of the immune system, is a (1,6)-branched (1,3)-β-glucan extracted from *L. edodes* fruiting bodies [5]. Our structure studies showed that Se-Le-30 fraction is a mixture of α-1,4-glucan and β-1,3-branched β-1,6-d-glucan with a much higher than lentinan molecular weight (3.62 × 10^6^ g/mol vs. 1.153 × 10^3^ g/mol). This may explain why the biological activity of the Se-Le-30 observed in the present study was different (i.e., opposite) to lentinan.

Our data reveal that Se-Le-30 significantly inhibits anti-CD3-, but not PHA- or SAC-stimulated proliferation of human PBMCs. These observations are consistent with our findings for T-cell proliferation measured in human allogeneic MLR. This outcome suggests that Se-Le-30 is a selective immunosuppressant, which acts through the modulation of signaling via the TCR-CD3 receptor complex; however, the exact mechanism of action was not examined at this stage of research. It is worth emphasizing that selective immunosuppressive activity is not typical for polysaccharides of fungal origin.

Next, we explored whether Se-Le-30 affects the cytokine production of human CD3+ T cells. Intracellular cytokine staining by flow cytometry demonstrated that the analyzed fraction significantly down-regulated the production of TNF-α; however, it had no effect on the numbers of IL-10-, IFN-γ- and TGF-β-producing CD3+ T cells. Opposite results were obtained in a few studies analyzing the biological activity of lentinan, which is considered an effective immunostimulatory drug [9,10]. Wang et al. [23] revealed that lentinan, in a dose-dependent manner, increased the anti-CD3 antibody-induced proliferation of T cells in mice. Moreover, numerous studies confirmed that this drug augments the production of some immune mediators, including IL-1α and IL-1β [24,25], IL-3 [26], IL-6 [27], IL-10, TGF-β1, IFN-γ, TNF-α, and IL-12 [25,28]. In addition, several reports suggested that lentinan stimulates the proliferation of lymphocytes, monocytes and macrophages [29]. We assume that opposite results obtained in our study and research for lentinan are associated with different structures of these glucans since the elements of the polysaccharide structure determine their biological activity [6].

In our study, we also investigated the effects of Se-Le-30 on human NK cells’ cytotoxic activity. These cells express complement receptor type 3 (CR3) and toll-like receptor 4 (TLR-4), which are recognized by polysaccharides [30]. We found that the analyzed fraction significantly down-regulated cytotoxic activity of human NK cells. In contrast to Se-Le-30, recent studies demonstrated that lentinan activates the NK cells of healthy blood donors and cancer patients. It was observed that the drug promoted the cytotoxicity of NK cells via the upregulation of IFN-γ and perforin production and increasing the expression of the activating receptor NKp30 [31,32]. In similar studies, the enhancement of NK activity was observed in chronic lymphocytic leukemia patients [33,34].

It has been demonstrated that receptors for β-glucans are expressed on neutrophils [35]. Moreover, as in our previous study, we showed that Se-enriched polysaccharides exert antioxidant effects in HeLa cells [16]. In the present study, we decided to analyze the effects of Se-Le-30 on the production of superoxide anions (O_2_^−^) using human peripheral blood granulocytes. However, we found no significant influence of Se-Le-30 on the reactive oxygen species production by these cells. This suggests that the antioxidant activity observed in the previous analysis is not related to the inhibition of ROS synthesis.

In summary, the results obtained in the present study indicate that Se-Le-30, an analog of lentinan, isolated by the modified Chihara method from the Se-enriched mycelium of *L. edodes*, is a T/NK cell-selective immunosuppressant, which is not typical for mushroom-derived β-glucans. Our results are, therefore, innovative and substantiate the necessity for further comprehensive investigations on the immunomodulatory activities of Se-Le-30 towards prospective applications as an immunosuppressive compound.

## 5. Patents

The result of the described research is a patent application P.438570 “Selenized bioactive polysaccharide fraction from *Lentinula edodes*, a pharmaceutical composition comprising selenized bioactive polysaccharide fraction, the selenized bioactive polysaccharide fraction for use as medicaments and a method of preparation thereof” filed with the Patent Office of the Republic of Poland on 22 July 2021.

## Data Availability

All the data are illustrated in the figures and in the Supplementary Materials.

## Supplementary Materials

The following are available online at https://www.mdpi.com/article/10.3390/biom11121777/s1, Figure S1: The structure of polysaccharides presented in fraction Se-Le-30 versus lentinan: (A) α(1→4) glucan; (B) β(1→3) glucan; (C) β(1→6) glucan; (D) β(1→3)-β(1→6) glucan, (E). β(1→6)-β(1→3) glucan (lentinan).Figure S2: Gating strategy for flow cytometry intracellular cytokine expression assay. Mononuclear cells were gated on the FSC-A/SSC-A plot as cells below 50x × 10^3^ on SSC and 50–200 × 10^3^ FSC (A). Data from previously gated population cell aggregates were excluded (B). Subsequently, CD3+ cells were identified by fluorescence above 10^3^ on the PerCP channel (C). An experiment was conducted in two variations of intracellular staining: IFN-γ-FITC and TGF-β-PE (Tube 1), TNFα-FITC and IL-10-PE (Tube 2). The cut-off value for positive fluorescence of studied intracellular cytokines (D1, D2, E1, E2) was based on the Fluorescence-Minus-One experiment and corresponding isotype controls. The hierarchy for studies populations and target cells (F1 and F2).
