# Supplementary material for: Selective Biological Effects of Selenium-Enriched Polysaccharide (Se-Le-30) Isolated from Lentinula edodes Mycelium on Human Immune Cells
Source: Biomolecules. 2021 Nov 26;11(12):1777. doi: 10.3390/biom11121777 (PMC8698628; doi:10.3390/biom11121777)
Supplement: Supplementary file 1 [file biomolecules-11-01777-s001.zip › biomolecules-1462807-supplementary.pdf]

A. [→4-α-D-Glc*p*-(1→4)-α-D-Glc*p*-(1→]$_n$ α(1→4)-glucan

B. [→6-β-D-Glc*p*-(1→6)-β-D-Glc*p*-(1→]$_n$ β(1→6)-glucan

C. [→3-β-D-Glc*p*-(1→3)-β-D-Glc*p*-(1→]$_n$ β(1→3)-glucan

D. [→6-β-D-Glc*p*-(1→6)-β-D-Glc*p*-(1→6)-β-D-Glc*p*-(1→]$_n$ β(1→3)-β(1→6)-glucan
3
↑
β-D-Glc*p*

E. [→3-β-D-Glc*p*-(1→3)-β-D-Glc*p*-(1→3)-β-D-Glc*p*-(1→]$_n$ β(1→6)-β(1→3)-glucan (Lentinan)
6
↑
β-D-Glc*p*

Supplementary Figure S1. The structure of polysaccharides presented in fraction Se-Le-30 versus lentinan: (A) *α*(1→4) glucan; (B) β(1→3) glucan; (C) β(1→6) glucan; (D) β(1→3)-β(1→6) glucan, (E). β(1→6)-β(1→3) glucan (lentinan).

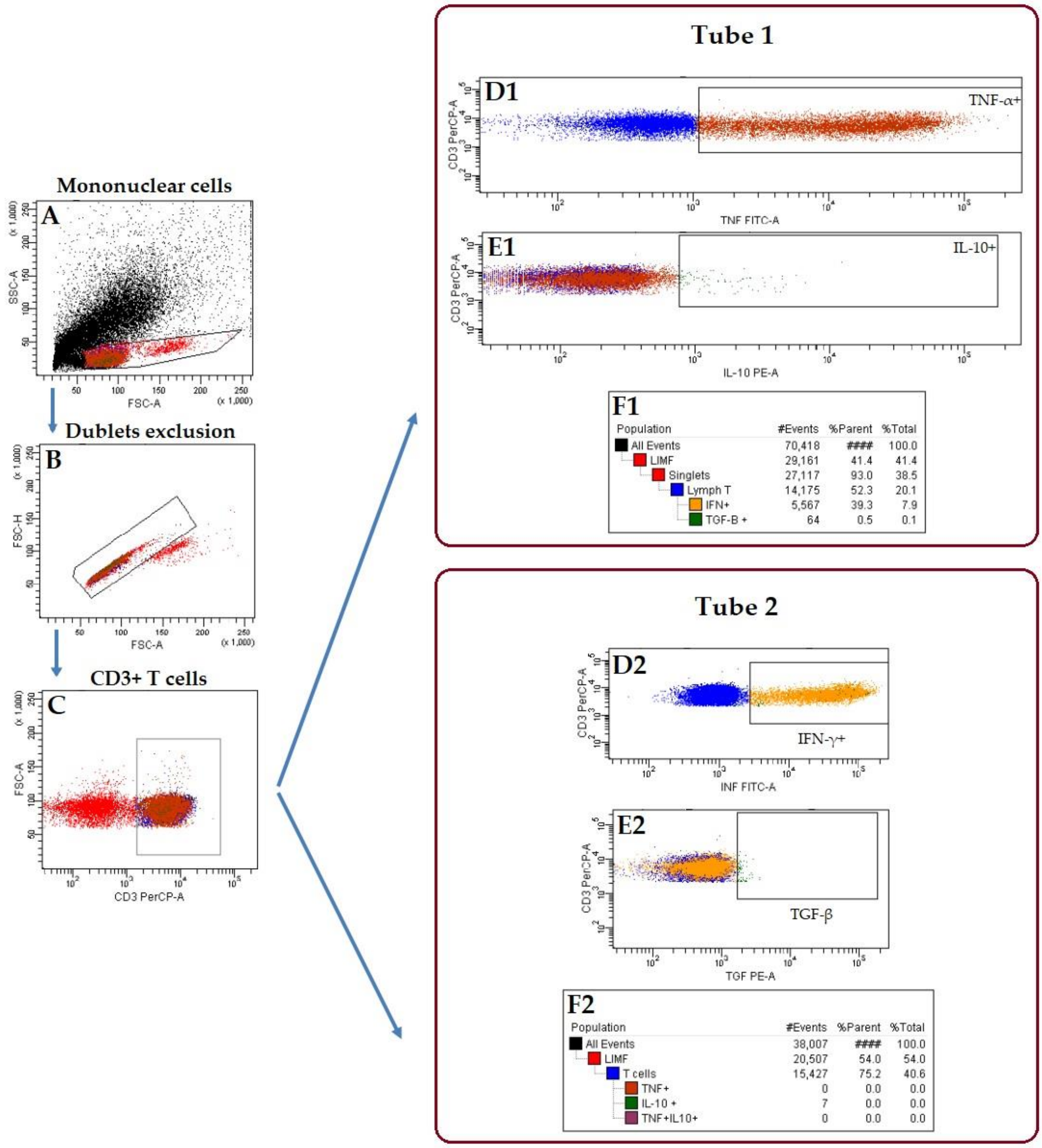

Supplementary Figure S2. Gating strategy for flow cytometry intracellular cytokine expression assay. Mononuclear cells were gated on FSC-A/SSC-A plot as cell below 50x10^3 on SSC and 50-200x10^3 FSC (A). From previously gated population cell aggregates were excluded (B). Subsequently CD3+ cells were identified by fluorescence above 10^3 on PerCP channel (C). An experiment was conducted in two wariations of intracellular staining: IFN-γ - FITC and TGF-β - PE (Tube 1), TNF-α - FITC and IL-10 - PE (Tube 2). Cut-off value for positive fluorescence of studied intrecellular cytokines (D1, D2, E1, E2) were based on Fluorescence-Minus-One experiment and corresponding isotype controls. The hierarchy for studies populations and target cells (F1 and F2).
